# Liraglutide in Adults With Obesity: A Multicentre, Single‐Arm, Post‐Marketing Surveillance Study in Taiwan

**DOI:** 10.1002/edm2.70333

**Published:** 2026-09-19

**Authors:** Shih‐Te Tu, Chiao‐Fan Chiu, Chu‐Kuang Chou, Hai‐Hua Chuang, Shu‐Wei Hu, Wen‐Ching Ko, Chun‐Ying Lee, Wen‐Yuan Lin, Yen‐Jen Sung, Thing‐Fong Tzeng, Weu Wang, Hsiang‐Fan Wu, Chun‐Yan Yeung, Bill Chen, Chet Joe Sim, Kuo‐Chin Huang

**Affiliations:** ^1^ Division of Endocrinology and Metabolism, Department of Internal Medicine Changhua Christian Hospital Changhua Taiwan; ^2^ Division of Pediatric Endocrinology and Genetics, Department of Pediatrics Linkou Chang Gung Memorial Hospital Taoyuan Taiwan; ^3^ School of Medicine, College of Medicine, Chang Gung University Taoyuan Taiwan; ^4^ Graduate Institute of Clinical Medical Sciences, College of Medicine, Chang Gung University Taoyuan Taiwan; ^5^ Division of Gastroenterology and Hepatology, Department of Internal Medicine Ditmanson Medical Foundation Chia‐Yi Christian Hospital Chiayi Taiwan; ^6^ Obesity Center, Ditmanson Medical Foundation Chia‐Yi Christian Hospital Chiayi Taiwan; ^7^ Department of Community Medicine Cathay General Hospital Taipei Taiwan; ^8^ School of Medicine, National Tsing Hua University Hsinchu Taiwan; ^9^ Department of Industrial Engineering and Management National Taipei University of Technology Taipei Taiwan; ^10^ Chia an Medical Clinic Taichung Taiwan; ^11^ Division of Pediatric Gastroenterology and Hepatology, Department of Pediatrics Tungs' Taichung MetroHarbor Hospital Taichung Taiwan; ^12^ Division of Adolescent Medicine, Department of Pediatrics Tungs' Taichung MetroHarbor Hospital Taichung Taiwan; ^13^ Department of General Surgery MacKay Memorial Hospital Taipei Taiwan; ^14^ Department of Family Medicine Kaohsiung Medical University Chung‐Ho Memorial Hospital Kaohsiung Taiwan; ^15^ Department of Family Medicine China Medical University Hospital Taichung Taiwan; ^16^ Department of Social and Family Medicine, School of Medicine China Medical University Taichung Taiwan; ^17^ Genesis Clinic Taipei Taiwan; ^18^ TZ Clinic Taipei Pingtung Taiwan; ^19^ Division of Digestive Surgery, Department of Surgery Taipei Medical University Hospital Taipei Taiwan; ^20^ Department of Surgery, School of Medicine College of Medicine, Taipei Medical University Taipei Taiwan; ^21^ Department of Pediatrics Kuang Tien General Hospital Taichung Taiwan; ^22^ Department of Pediatrics Hsinchu Municipal MacKay Children's Hospital Hsinchu Taiwan; ^23^ Department of Medicine MacKay Medical University New Taipei Taiwan; ^24^ Clinical, Medical and Regulatory Novo Nordisk Taiwan; ^25^ Medical Affairs Novo Nordisk Taiwan; ^26^ Department of Family Medicine National Taiwan University Hospital Taipei Taiwan; ^27^ Department of Family Medicine College of Medicine, National Taiwan University Taipei Taiwan

**Keywords:** GLP‐1 analog, obesity, real‐world evidence

## Abstract

**Introduction:**

This prospective study (NCT06283641) was the first real‐world, multicentre, post‐marketing surveillance of GLP‐1 receptor agonist liraglutide as an adjunct for weight management in adults with obesity in Taiwan.

**Methods:**

Data were collected from 14 sites at baseline, week 13 and week 26 (February 2024–January 2025). Safety and effectiveness outcomes, including adverse event (AE) incidence proportions (IPs) and body weight (BW) change, were investigated.

**Results:**

Among 259 adults, 61.0% were female (mean ± SD age 42.6 ± 11.3 years, BW 90.4 ± 17.6 kg, BMI 33.0 ± 4.7 kg/m^2^). Dyslipidaemia (38.2%), hypertension (26.3%), and diabetes (21.2%) were common. Mean daily and maximum doses were 1.4 ± 0.5 and 1.8 ± 0.7 mg/day, respectively. Overall IP of AEs was 11.2%; 90.9% of events were mild. General/injection‐site and gastrointestinal events were most frequent. Medication cost (49.0%) was the predominant factor for dose non‐escalation. At weeks 13 and 26, mean BW loss was −5.7 ± 3.5 and −8.5 ± 5.7 kg (both *p* < 0.0001; ≥ 5% BW loss in 58.4% and 76.7%) and mean systolic blood pressure decrease was 9.0 ± 16.7 and 10.4 ± 18.9 mmHg, respectively.

**Conclusion:**

Liraglutide at < 3.0 mg/day was well‐tolerated, with BW reduction observed over 26 weeks in Taiwanese adults. Medication cost is a barrier to long‐term obesity management and a significant consideration for health policies in Taiwan.

## Graphical abstract

In this multicentre real‐world post‐marketing surveillance (rPMS) study conducted in Taiwan, reductions in body weight and no new safety issues were observed among adults with obesity treated with liraglutide.

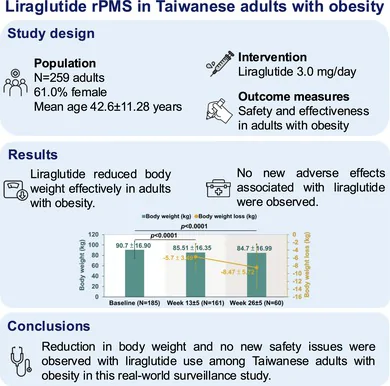

## 1 Introduction

Obesity is a significant global public health challenge with considerable health impacts [1]. The prevalence of adult obesity in Taiwan has markedly increased in recent years from 11.8% in 1993–1996 to 23.9% in 2017–2020 [2, 3], which poses a higher risk of developing obesity‐related comorbidities, such as hypertension, Type 2 diabetes mellitus (T2DM) and atherosclerosis [4]. Importantly, the clinical burden of obesity varies by race and ethnicity. Individuals of Asian descent experience a higher prevalence of metabolic comorbidities, such as T2DM and cardiovascular disease, at a lower body mass index (BMI) than individuals of European descent [5, 6]. Consequently, the World Health Organisation guidelines recognise lower BMI thresholds for overweight (≥ 23.0 kg/m^2^) and obesity (≥ 25.0 kg/m^2^) in Asians and Pacific Islanders [1]. On the other hand, Taiwan has adopted obesity and overweight thresholds of ≥ 27.0 kg/m^2^ and ≥ 24.0 kg/m^2^, respectively, to better reflect local epidemiological data and clinical context [2, 7]. Furthermore, organisations including the American Medical Association, National Institutes of Health, The Obesity Society and the American Association for Clinical Endocrinology have classified obesity as a disease [8], indicating the gravity of obesity and related comorbidities. This recognition highlights the necessity for further improvements in preventive, diagnostic and treatment strategies for obesity. Enhancing weight loss remains a primary focus in obesity management, as weight reduction offers a broad range of significant health benefits such as optimising glycaemic control and mitigating progression to T2DM, lowering blood pressure (BP) and cholesterol levels, and improving sleep apnoea [9, 10, 11].

Liraglutide is a once‐daily glucagon‐like peptide‐1 (GLP‐1) analogue with 97% homology to human GLP‐1 [12]. Eating stimulates the release of multiple hormonal and neuronal signals, including GLP‐1 and other gut hormones, which are subsequently processed by the brain and translated into hunger or satiety to regulate food intake [13]. The effects of liraglutide on feeding and metabolic physiology are mediated via specific activation of the GLP‐1 receptor in brain regions that are critical to the regulation of energy intake, such as the hypothalamus and amygdala [14]. In both animal and human studies, peripheral administration of liraglutide led to decreased food intake and weight loss [14, 15, 16].

Similar to other GLP‐1 receptor agonists (GLP‐1 RAs), liraglutide delays gastric emptying [12, 17, 18, 19], which may modestly contribute to reductions in postprandial glucose. Moreover, liraglutide stimulates insulin secretion, reduces inappropriately high glucagon secretion, and improves pancreatic beta‐cell function [12, 17]. These physiologic changes lead to decreased fasting and postprandial glucose levels and increased energy expenditure, subsequently resulting in weight loss via a reduction in fat mass [20]. Liraglutide represents an important therapeutic option for obesity, as it regulates body weight in addition to blood glucose levels [21].

In all five adult clinical studies in the global weight management program SCALE (Satiety and Clinical Adiposity—Liraglutide Evidence in individuals with and without diabetes) [22, 23, 24, 25, 26], liraglutide 3.0 mg as an adjunct to a reduced‐calorie diet and increased physical activity induced a mean weight loss > 5% from baseline weight, superior to that achieved with lifestyle modification alone. Similar weight reductions were observed in other studies conducted in Europe and Asia [27, 28]. The safety profile of liraglutide 3.0 mg used for weight loss in adults was consistent with that observed with liraglutide at 1.8 mg for managing diabetes [23, 25, 26], with transient gastrointestinal (GI) events reported most frequently. However, randomised controlled trials (RCTs) like the SCALE program operate under highly controlled conditions with strict titration schedules and monitoring and often exclude patient populations with complex comorbidities. Thus, the efficacy data from RCTs may not fully translate to real‐world clinical practice, where treatment adherence, dosing strategies, and treatment tolerability vary across different cohorts.

Despite the rapid rise of obesity in Taiwan, real‐world data regarding newer anti‐obesity medications (AOMs) remain limited. Liraglutide was approved for adult obesity treatment in Taiwan in 2020 [29] before other similar agents and is currently the most widely available incretin‐based AOM for clinical use in Taiwan. Thus, we selected liraglutide to assess the real‐world effectiveness of GLP‐1 RAs for treating obesity in Asians and Pacific Islanders [1]. To address critical gaps in current literature, this study aims to identify variations in real‐world dosing patterns (including sub‐maximal maintenance doses), weight loss effectiveness and incidence of adverse events (AEs) under routine clinical surveillance compared to data from other countries and ethnicities. This post‐authorisation study utilised pragmatic inclusion and exclusion criteria to determine a realistic benchmark of liraglutide therapy in the Taiwanese adult population.

## 2 Materials and Methods

### 2.1 Study Design and Participants

This study was a multi‐centre, single‐arm, non‐interventional study sponsored by Novo Nordisk Pharma Taiwan, Ltd. to evaluate the safety and effectiveness of liraglutide in adult patients with obesity in Taiwan from February 2024 to January 2025. Patients ≥ 18 years of age were recruited from 14 hospitals and clinics if they (a) were scheduled to receive liraglutide treatment according to approved label use or had initiated liraglutide treatment within 7 days of recruitment, and (b) had baseline data (body height, weight and initial dosage of liraglutide) available. All participants decided to undergo treatment with liraglutide independently of the decision to join this study and were provided informed consent before data collection. Exclusion criteria included known or suspected hypersensitivity to liraglutide or any of its excipients; previous participation in this study; treatment with an investigational drug within 30 days prior to initiating liraglutide treatment; female patient who was pregnant, breast‐feeding, or intending to become pregnant; personal or family history of medullary thyroid cancer or multiple endocrine neoplasia Type 2; and mental incapacity, unwillingness or language barriers precluding adequate understanding or cooperation. The planned number of subjects to be enrolled was 248 adults; 172 participants were estimated to receive treatment and complete at least one post‐treatment visit for inclusion in the safety analysis. The sample size calculation is as follows:

$$\mathrm{Pr}\;\left(X\geq 1\right)=1–\mathrm{Pr}\;\left(X=0\right)$$

$$N=\frac{-\ln\;\left(1-\mathrm{Pr}\;\left(X\geq 1\right)\right)}{\lambda }\;\left(\lambda :\text{incidence}\right)$$

$$N=\frac{3}{\lambda }=\frac{3}{0.015}=200$$

Information regarding the study, including involved procedures, patient rights, and possible advantages and disadvantages of participating in the study, was provided in writing and verbally explained to eligible patients. Participants voluntarily completed a signed and dated informed consent form prior to any activity or evaluation related to the study and were continuously enrolled in the study until the planned number of enrolled patients was reached. This study was approved by relevant institutional review boards and independent ethics committees in Taiwan and adhered to the requirements outlined in the Declaration of Helsinki [30] and Good Pharmacoepidemiology Practice guidelines [31].

Following informed consent, relevant participant demographic information was collected. Participant body weight, vital signs, and the initial dose, dose adjustment, and safety profiles of liraglutide were collected at the time of medication initiation (baseline), week 13, and week 26. Participants were allowed to withdraw from the study at any time. Any visit at which participants chose to withdraw from the study was considered an early termination visit, with reasons documented. Appropriate measures to protect the identity of study participants, as required by local, regional, and national regulations, were taken.

### 2.2 Dose Administration, Exposure and Use of Liraglutide

Dosage and duration of treatment were determined by prescribing physicians according to instructions on the Saxenda Instruction Insert Taiwan [29]. Treatment was initiated with 0.6 mg of liraglutide and subsequently escalated by 0.6 mg each week if the prior dose was well‐tolerated until the recommended maintenance dose of 3.0 mg was reached. Dose escalation may be delayed by one week if the patient was unable to tolerate the present dose [29, 32]. Participants remained on their maximum tolerated dose until study completion or discontinuation.

Receipt of liraglutide for 13 weeks or longer was categorised as ‘long‐term use’ regardless of dose. ‘Long‐term use’ was used as an operational term specific to this study to distinguish participants who continued treatment beyond the first scheduled follow‐up assessment at Week 13 from those who discontinued treatment before completing 13 weeks of treatment. This definition should not be interpreted as long‐term management from the conventional clinical perspective.

### 2.3 Variables

The primary endpoint was the incidence of adverse events (AEs), assessed at week 13 and week 26. The secondary endpoint comprised further safety assessment (including adverse drug reactions [ADRs], serious AEs [SAEs], serious ADRs [SADRs], unexpected AEs, unexpected ADRs, unexpected SAEs, and unexpected SADRs) and effectiveness assessment at weeks 13 and 26.

Information on the safety of liraglutide was collected on NIS safety forms at the pre‐defined timepoints (week 13 and week 26), including each occurrence of an AE, AE preferred term, start and stop dates, seriousness, severity, causal relationship to liraglutide, actions taken with liraglutide, and outcomes of the AE. All AEs were considered adverse drug reactions (ADRs) unless assessed as ‘unlikely’ in the causality assessment. Events were categorised using the Medical Dictionary for Regulatory Activities version 26.1 by system‐organ class and preferred term (PT) for the safety assessment.

The dose and administration duration of liraglutide after initiation, and any reasons for not escalating the dose to 3.0 mg for maintenance as specified in the product label were also recorded in the safety assessment. Data for all enrolled participants were included in the safety analysis.

The effectiveness assessment evaluated the percentage (%) and absolute value (kg) of body weight loss from baseline to week 13 and week 26; the proportion of subjects who lost at least 5% of baseline body weight at week 13 and week 26; and the proportion of subjects who lost at least 10% of baseline body weight at week 13 and week 26. Patients who withdrew from the study before week 13 or before week 26 were excluded from the effectiveness analysis for the respective timepoint.

### 2.4 Statistical Analysis

Participant demographics and baseline characteristics were summarised with number (N) and percentage (%), mean ± standard deviation (SD) or median (minimum [min], maximum [max]). Liraglutide dosing and the length of the administration period for each subject were described as frequency distributions or basic summary statistics.

AEs were summarised with the number of events, the number of participants with the AE, an incidence proportion (IP) (%), an incidence rate (IR) per 100 patient year of exposure (PYE) and its 95% CI, and an IR per 100 patient‐month of exposure (PME) and its 95% CI. ADRs, SAEs/SADRs, unexpected AEs/ADRs and unexpected SAEs/SADRs were summarised with the number of events, number of subjects with events and IP (%). IPs and IRs per 100 PYE or PME were calculated with the following formulas.

IP = (Number of subjects with AE)/(Number of subjects in safety analysis set) × 100 (%).

IR per 100 PYE = (Number of subjects with AE)/((Total administration period of subjects in safety analysis set)/365 days) × 100.

IR per 100 PME = (Number of subjects with AE)/((Total administration period of subjects in safety analysis set)/30 days) × 100.

Effectiveness was assessed by per‐protocol analysis. The percentages (%) of body weight loss from baseline to week 13 and to week 26 were analysed using ANOVA (≥ 3 groups) or *t*‐test between subgroups in an exploratory analysis of the impact of liraglutide on body weight. Absolute values (kg) of body weight loss from baseline to week 13 and to week 26 were analysed using ANCOVA with baseline weight as covariate. This approach was selected to assess the treatment effect at specific endpoints rather than to model its temporal trajectory.

Weight loss percentage and absolute value were described with statistics for mean body weight change at both time points. The proportions of subjects who lost ≥ 5% and ≥ 10% of their baseline body weight were described with the percentage (%) and 95% CI of participants and the average percentage (%) of weight loss at week 13 and week 26.

No imputations were considered for missing data in the analysis.

## 3 Results

We evaluated 259 adult participants receiving liraglutide under routine clinical care in Taiwan. The demographics and baseline characteristics of the study population are described in Table 1. More women participated in this study (158/259, 61%), and the mean age of all participants was 42 years. Hypertension was a prevalent comorbidity in this study population, with the mean systolic blood pressure (SBP) at 134.8 ± 17.0 mmHg.

**TABLE 1:** Demographics and baseline characteristics of adults treated with liraglutide.

Demographic	*N* (%)^a^ (*N* = 259)
Sex	
Female	158 (61.0%)
Age (years)	
Mean ± SD	42.6 ± 11.3
Median (min, max)	42.0 (21, 67)
Age Group	
20 to 29 years	33 (12.7%)
30 to 39 years	75 (29.0%)
40 to 49 years	78 (30.1%)
50 to 59 years	52 (20.1%)
≥ 60 years	21 (8.1%)
Weight (kg)	
Mean ± SD	90.4 ± 17.6
Median (min, max)	87.4 (58.0, 162.1)
BMI (kg/m^2^)	
Mean ± SD	33.0 ± 4.7
Median (min, max)	32.1 (27.0, 52.3)
SBP (mmHg)	
Mean ± SD	134.8 ± 17.00
Median (min, max)	132.0 (98, 194)
DBP (mmHg)	
Mean ± SD	82.3 ± 11.6
Median (min, max)	81.0 (58, 135)
Concomitant AOM use^b^	131 (50.6%)
Medical history	
Dyslipidaemia	99 (38.2%)
Hepatic impairment	71 (27.4%)
Hypertension	68 (26.3%)
Diabetes	55 (21.24%)
Gastroesophageal reflux disease	32 (12.4%)
Sleep apnoea syndrome	11 (4.3%)

Abbreviations: AOM, anti‐obesity medication; BMI, body mass index; DBP, diastolic blood pressure; max, maximum; min, minimum; SBP, systolic blood pressure; SD, standard deviation.

^a^ Values in N (%) unless otherwise specified.

^b^ AOM included repurposed use of diabetes medications.

**FIGURE 1:**
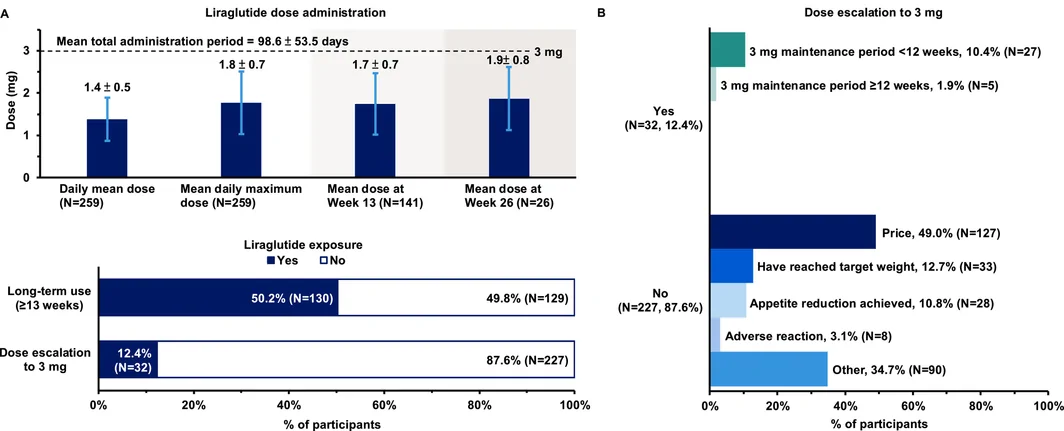
Dose administration and exposure in adults treated with liraglutide. (A) The mean daily administration dose of liraglutide (mean ± SD) was 1.4 ± 0.5 mg/day. The mean maximum daily dose was 1.8 ± 0.7 mg/day. Mean doses at week 13 and week 26 were 1.7 ± 0.7 mg and 1.9 ± 0.8 mg. The mean total administration period was 98.6 ± 53.5 days. 130/259 participants (50.2%) received liraglutide treatment for ≥ 13 weeks, and 32/259 participants (12.4%) escalated their maximum dose to 3.0 mg. *N* = 141 and *N* = 26 indicate the number of participants who completed 13 weeks and 26 weeks of liraglutide treatment, respectively. (B) 127/227 (49.0%) of the participants who did not escalate their dosage to 3.0 mg reported medication price as their primary reason, whereas 8/227 participants (3.1%) cited adverse reactions.

The statistics of liraglutide dose administration and exposure are summarised in Figure 1. Over the mean total administration period of approximately 14 weeks, the mean daily and weekly doses were well below the recommended target dose of 3.0 mg. Nearly half of participants (118/259, 45.6%) withdrew before week 13, raising concerns about potential attrition bias in effectiveness analysis. A summary of participant disposition is shown in Table A1. 130/259 (50.2%) completed long‐term treatment (≥ 13 weeks), and 26/259 participants (10.0%) who were treated with liraglutide long‐term received treatment for 26 weeks. Among participants who did not escalate to 3.0 mg (*n* = 227), 127 (49.0%) cited medication cost as the main reason.

Reported AEs and ADRs and their respective incidences are described in Table 2. GI events, general disorders and administration site conditions were the most common AEs (IP = 4.3% and 2.3%, respectively). Low IPs of unexpected AEs, SAEs and unexpected SAEs (data not shown), and no SADRs were documented. The most common unexpected AE encountered was infections and infestations (6 cases/259, 2.3%), specifically COVID‐19 (3 cases/259, 1.2%), with most (4/6) patients continuing dose administration. Obstructive sleep apnoea and benign hepatic neoplasm each occurred in one participant (0.4%) as SAEs and unexpected SAEs. We further analysed the IP of AEs between subgroups (sex, age group, long‐term use, medical history, hepatic impairment and concomitant AOM use) and did not find statistical significance with this stratification.

**TABLE 2:** Incidence of adverse events reported among adults treated with liraglutide.

	Number (%) of patients (*N* = 259)	Number of events (E = 44)
Total Number of AEs	29 (11.2%)	44
Before 13 weeks	29 (11.2%)	42
After 13 weeks	2 (0.8%)	2
Severity		
Mild		40
Moderate		3
Severe		1
IP of AEs		
General disorders and administration site conditions	11 (4.3%)	14
Injection site erythema	4 (1.5%)	4
Injection site dermatitis	3 (1.2%)	3
Injection site hypersensitivity	2 (0.8%)	2
Injection site pruritus	2 (0.8%)	2
Injection site rash	1 (0.4%)	1
Fatigue	1 (0.4%)	1
Swelling	1 (0.4%)	1
Gastrointestinal disorders	6 (2.3%)	8
Constipation	3 (1.2%)	3
Nausea	2 (0.8%)	2
Abdominal distension	1 (0.4%)	1
Diarrhoea	1 (0.4%)	1
Stomatitis	1 (0.4%)	1
Nervous system disorders	6 (2.3%)	7
Dizziness	4 (1.5%)	5
Headache	1 (0.4%)	1
Sciatica	1 (0.4%)	1
Infections and infestations	6 (2.3%)	6
COVID‐19	3 (1.2%)	3
Influenza, pneumonia, sinusitis	3 (1.2%)	3
Other		
Ligament sprain, skin abrasion	2 (0.8%)	2
Cough, obstructive sleep apnoea	2 (0.8%)	2
Pruritus, rash	2 (0.8%)	2
Palpitations	1 (0.4%)	1
Benign hepatic neoplasm	1 (0.4%)	1
Haematuria	1 (0.4%)	1
IR of AEs per 100 PYE (95% CI)		41.4 (27.7, 59.4)
IR of AEs per 100 PME (95% CI)		3.4 (2.3, 4.9)
IP of ADRs	18 (7.00%)	25
General disorders and administration site conditions	10 (3.9%)	13
Injection site erythema	3 (1.2%)	3
Injection site dermatitis	3 (1.2%)	3
Injection site hypersensitivity	2 (0.8%)	2
Injection site pruritus	2 (0.8%)	2
Injection site rash	1 (0.4%)	1
Fatigue	1 (0.4%)	1
Swelling	1 (0.4%)	1
Gastrointestinal disorders	5 (1.9%)	6
Constipation	3 (1.2%)	3
Nausea	2 (0.8%)	2
Abdominal distension	1 (0.4%)	1
Nervous system disorders	3 (1.2%)	4
Dizziness	3 (1.2%)	4
Other		
Pruritus, rash	2 (0.8%)	2

Abbreviations: ADR, adverse drug reaction; AE, adverse event; CI, confidence interval; IP, incidence proportion; IR, incidence rate; PME, patient‐month of exposure; PYE, patient year of exposure.

We also assessed the effectiveness of liraglutide in facilitating weight loss (Table 3). In participants with long‐term use of liraglutide (≥ 13 weeks), we identified significant body weight reduction at both week 13 (mean −5.7 kg, *p* < 0.0001) and at week 26 (mean −8.5 kg, *p* < 0.0001). 94 of the 161 participants (58.4%) treated for 13 ± 5 weeks and 46 of the 60 participants (76.7%) treated for 26 ± 5 weeks achieved ≥ 5% body weight loss. Notably, 23 of the 60 participants (38.3%) treated for 26 weeks of treatment achieved > 10% weight loss.

**TABLE 3:** Changes in body weight between baseline, week 13 and week 26 among adults treated with liraglutide.

	Baseline (*N* = 185)	Week 13 ± 5 (*N* = 161)^a^	Week 26 ± 5 (*N* = 60)^a^
Body weight mean ± SD (kg)	90.7 ± 16.9	85.5 ± 16.4	84.7 ± 17.0
Body weight median (min, max) (kg)	87.4 (64.9, 162.1)	82.4 (57.5, 155.5)	82.9 (56.9, 149.3)
Body weight loss (kg)	Mean ± SD (kg)	−5.7 ± 3.5	−8.5 ± 5.7
	Median (min, max) (kg)	−5.5 (−23.6, 4.0)	−7.3 (−29.2, 1.6)
	*p*‐value for difference	*p* < 0.0001	*p* < 0.0001
Body weight loss (%)	Mean ± SD (kg)	−6.3 ± 3.7	−9.1 ± 5.8
	Median (min, max) (kg)	−6.1 (−21.4, 3.8)	−7.9 (−24.5, 1.9)
Subjects with weight loss ≥ 5%	% of subjects (95% CI)	58.4 (50.4, 66.1)	76.7 (64.0, 86.6)
	Average weight loss (%)	−8.5	−11.1
Subjects with weight loss ≥ 10%	% of subjects (95% CI)	9.9 (5.8, 15.6)	38.3 (26.1, 51.8)
	Average weight loss (%)	−14.3	−15.1

Abbreviations: CI, confidence interval; max, maximum; min, minimum; SD, standard deviation.

^a^ *N* = 161 and *N* = 60 indicate the number of participants in the effectiveness analysis who initiated 13 or 26 weeks of treatment, respectively, including those who did not complete treatment for the entire week.

## 4 Discussion

This study demonstrated that liraglutide was well tolerated and effective at lower doses for weight loss in adults in Taiwan. Consistent with patterns observed in other studies, the most common AEs in this study were GI and administration‐site events [21, 23, 24, 28, 33]. However, there were some variations in the incidence of AEs between this and other investigations. The overall IR of AEs was 41.4 per 100 PYE, markedly lower than the 321.8 per 100 PYE reported in a RCT [23]. However, differences in dose, study duration and reporting methods may limit direct comparability. The IP of GI AEs in this study was lower than those from earlier trials (1.2–1.8 mg, IP = 53.7%–60.0% [28]; IP = 20.8%, 2.5% and 2.1% for nausea, diarrhoea and constipation, respectively [33]), and no GI AEs progressed to SAEs such as cholelithiasis, cholecystitis or pancreatitis. The incidence of general disorders and injection site events we observed (IP = 4.3%) was likely similar to those seen in previous investigations. One RCT reported injection‐site haematoma and fatigue occurring at 6.9 and 9.1 events per 100 PYE, respectively [23], although another trial documented higher IPs of general disorders and administration site conditions (1.2–1.8 mg, 16.8%–17.8%) [28].

The lower IP of AEs and SAEs, particularly GI disorders, documented in this study may be attributable to the lower liraglutide dose used (mean ± SD, 1.4 ± 0.5 mg/day). An exposure‐response analysis similarly noted that larger doses of liraglutide correlated with a higher number of all nausea and vomiting events [34]. Real‐world treatment exposure may deviate from the recommended protocol based on physician judgement due to factors including patient tolerance of the drug and treatment affordability. In addition, real‐world dosing patterns may be adjusted at the clinician's discretion to follow more flexible and slower titration schedules, in contrast to the fixed, rapid titration protocols in RCTs. Patients who followed adjusted titration schedules potentially developed better tolerance to AEs associated with liraglutide. Exclusion of AEs that are more commonly symptomatic only in later, more severe stages (e.g., pancreatitis) due to the shorter duration of this study (≤ 26 weeks) compared to previous trials (52–56 weeks) [21, 23, 24] may be another possible cause.

Additionally, patient attitudes toward reporting AEs [35], such as unwillingness to disclose mild events or a lack of knowledge about pharmacovigilance, may have resulted in under‐documentation of AEs in this study. Observational studies, such as the present investigation, rely on spontaneous reporting and less rigid medical record charting than RCTs, which may also have contributed to the under‐documentation of AEs among participants. Finally, the large proportion of participants discontinuing liraglutide treatment before reaching the halfway point (13 weeks) of the study may have led to an under‐representation of transient AEs in the final results.

Further data analyses comparing the frequency of injection‐site AE occurrence between this and previous trials across multiple time points may be needed to investigate whether patients using liraglutide develop tolerance to administration‐site conditions. In existing case reports on injection‐site delayed‐type hypersensitivity associated with liraglutide, patients discontinued liraglutide treatment after the occurrence of the AE or switched to a different GLP‐1 RA [36, 37, 38, 39, 40]. Whether tolerance to administration‐site hypersensitivity can be developed still warrants investigation. Finally, a subgroup analysis we included in the safety assessment found no significant differences between the subgroups (data not shown), indicating the safety of liraglutide across adults regardless of sex, age, comorbidities, and concomitant use of other AOMs.

This surveillance study supported the effectiveness of liraglutide for weight loss in adults with obesity, although many participants used doses below the recommended maintenance dose of 3.0 mg for shorter durations than in RCTs and other real‐world surveillance studies [22, 23, 24, 25, 26, 41, 42]. Our findings aligned with prior studies investigating the effectiveness of liraglutide for weight loss in Asian patients, which similarly found clinically significant weight reduction with submaximal doses administered over shorter treatment periods [27, 33, 43]. This observation has several potential explanations, including a greater proportion of female participants in the study (61.0%) and a lower proportion of participants with diabetes (21.24%) compared to other comorbidities (Table 1). Prior studies have demonstrated a greater effectiveness of liraglutide in decreasing waist circumference in women than in men, and the weight loss effects of liraglutide have been observed to be moderately lower in patients with diabetes compared with patients without diabetes [23, 24, 28, 41, 42]. However, we did not observe a statistically significant difference in weight loss between female and male participants (Table A2), and no comparative statistical analyses of weight loss by medical history subgroups, including diabetes, were conducted due to the limited sample size. Additionally, in our subgroup analysis comparing weight loss across age groups, we found no significant differences (Table A3). In future real‐world studies, age‐group analyses may be conducted using broader age ranges and fewer comparison groups to determine whether age affects the effectiveness of liraglutide for weight loss. Other subgroup analyses to consider include stratification by comorbidities and by baseline BMI and waist circumference. Furthermore, participants may have used lower doses due to treatment cost, which would cause weight loss but at a slower rate [42].

RCTs of liraglutide for obesity management reported GI AEs (e.g., nausea, vomiting) as the most common reason for discontinuing or not escalating to a higher dose of the medication [23, 27, 28]. In contrast, participants in this study who did not increase their daily dose (49.0%) or discontinued treatment (data not shown) cited medication cost as the most common reason. This finding highlights the need for health economic and policy measures, such as insurance coverage or subsidy adjustments, to improve long‐term access to GLP‐1 RAs. A recent modelled projection of obesity‐related health expenditures and economic burden (e.g., sick leave, disability or death) indicated that GLP‐1 RAs are likely to decrease lifetime total economic costs in individuals with obesity [44]. However, another model comparing the cost projections of liraglutide 1.8 mg and once‐weekly semaglutide 1 mg in the UK health system found semaglutide to be more cost‐effective for management of chronic metabolic conditions, even if the cost of liraglutide were to be reduced [45]. Similarly, a cost‐effectiveness study comparing subcutaneous tirzepatide, oral semaglutide, subcutaneous semaglutide and subcutaneous liraglutide use in the U.S. for obesity management in adults without T2DM noted tirzepatide and oral semaglutide to be more cost‐effective [46].

Nonetheless, AOM selection should be individualised to the comorbidities and AE tolerability of each patient, and medication costs should therefore be adjusted for all GLP‐1 RAs approved for weight loss in Taiwan via methods such as optimised National Health Insurance subsidies. Cost adjustment for an effective and versatile medication (e.g., liraglutide for managing both diabetes and obesity) to expand its affordability and reduce socioeconomic burden is a complex challenge that deserves a focused dialogue with perspectives from health economics and other areas of expertise beyond the scope of this discussion. Given the obesity epidemic and rising prevalence of T2DM in adults in Taiwan [3], the affordability of medications that target both conditions and are well‐tolerated by patients (as indicated by the low number of participants who withdrew due to AE intolerance), such as liraglutide, is an urgent topic for the healthcare and biomedical research sectors to address.

At weeks 13 and 26, we also observed a moderate decrease in BP, with a more pronounced drop in systolic BP, corroborating findings from earlier liraglutide studies [23, 24, 27, 28]. Physiologic studies have suggested increased urinary sodium excretion [47, 48] due to renal GLP‐1 receptor activation [49] as a potential mechanism behind liraglutide‐associated BP reduction. This trend in BP changes is worth following in future studies of liraglutide in real‐world populations, as it suggests potential uses for liraglutide and other GLP‐1 RAs in cardiovascular comorbidities associated with chronic metabolic conditions, such as coronary artery disease. Given that the mean BP observed in this study was higher than that in previous trials [23, 28], liraglutide holds potential for incorporation into hypertension medication regimens in adults with obesity in Taiwan.

This study had several limitations. Because the study did not include a control group, our observations should be interpreted as confirmatory data rather than as evidence of causal relationships between liraglutide and the observed outcomes. The methodology was designed to assess outcomes only in participants who adhered to the treatment and completed follow‐up visits at the specified endpoints and did not conduct imputations for missing data, as the primary objective of this study was to evaluate the safety and effectiveness of liraglutide in real‐world clinical practice. The findings are underpowered to detect rare, serious AEs, as only 44 events were reported. Our classification of AEs may have overestimated ADR frequency, as all AEs were presumed ADRs unless explicitly assessed as unlikely. Many participants did not escalate their liraglutide maintenance dose to 3.0 mg as recommended, which may have limited the observed effectiveness and confounded the lower incidence and seriousness of AEs observed in this study. Lifestyle intervention was not standardised; thus, its potential impact on the study outcomes could not be evaluated, which might further confound the effectiveness results. Moreover, the interval for each follow‐up timepoint (±5 weeks) encompassed a relatively wide time range, reflecting the real‐world nature of the study conducted within routine clinical practice, potentially introducing bias to the safety and effectiveness assessment results. The effectiveness of liraglutide in long‐term weight management could not be assessed due to the short study duration, and the per‐protocol analysis approach may have led to an overestimation of treatment effectiveness. Finally, a large proportion of participants discontinued treatment prior to week 13 of the study, which may have introduced attrition bias into the effectiveness and safety outcomes of liraglutide use for weight loss. Participant characteristics for those who discontinued liraglutide before reaching 13 weeks of treatment were not compiled, further limiting the generalisability of long‐term outcomes observed in this study.

Despite these limitations, this study made several important contributions. The effectiveness and safety of submaximal doses of liraglutide for obesity treatment were comparable to those at 3.0 mg found in previous trials. These results suggest that submaximal liraglutide doses are promising for achieving clinically significant weight loss while reducing the occurrence of AEs and minimising the financial burden of receiving treatment [50]. The low retention rate of participants for long‐term liraglutide treatment due to medication cost emphasises the crucial need for a review of health financing approaches for obesity pharmacotherapy. Reductions in blood pressure were also found in this study, indicating potential additional benefits that liraglutide may provide in the management of chronic metabolic conditions.

In this follow‐up study of liraglutide for weight loss in a real‐world adult population in Taiwan, body weight reduction was observed over 26 weeks of treatment at doses of < 3.0 mg/day, and no new safety issues or adverse drug reactions were found.

## Author Contributions

**Shih‐Te Tu:** investigation, writing – review and editing, supervision, resources. **Chiao‐Fan Chiu:** investigation, writing – review and editing, resources, supervision. **Chu‐Kuang Chou:** investigation, writing – review and editing, supervision, resources. **Hai‐Hua Chuang:** investigation, writing – review and editing, supervision, resources. **Shu‐Wei Hu:** investigation, writing – review and editing, supervision, resources. **Wen‐Ching Ko:** investigation, writing – review and editing, supervision, resources. **Chun‐Ying Lee:** investigation, writing – review and editing, supervision, resources. **Wen‐Yuan Lin:** investigation, writing – review and editing, supervision, resources. **Yen‐Jen Sung:** investigation, writing – review and editing, supervision, resources. **Thing‐Fong Tzeng:** investigation, writing – review and editing, supervision, resources. **Weu Wang:** investigation, writing – review and editing, supervision, resources. **Hsiang‐Fan Wu:** investigation, writing – review and editing, supervision, resources. **Chun‐Yan Yeung:** investigation, writing – review and editing, supervision, resources. **Bill Chen:** funding acquisition, writing – review and editing, project administration, conceptualization, methodology. **Chet Joe Sim:** project administration, writing – review and editing, funding acquisition, conceptualization, methodology. **Kuo‐Chin Huang:** investigation, writing – review and editing, supervision, resources.

## Funding

This study was supported by funding from Novo Nordisk Pharma Taiwan, Ltd. (study ID: NN8022‐7780).

## Conflicts of Interest

The authors declare no conflicts of interest.

## Data Availability

The data that support the findings of this study are available on request from the corresponding author. The data are not publicly available due to privacy or ethical restrictions.

## Appendix

**TABLE A1:** Participant disposition.

Disposition	Number (%) of participants (*N* = 259)
Study Completion	
Completed Study	61 (23.55%)
Did Not Complete the Study	198 (76.45%)
Treatment Discontinuation: No	36 (13.90%)
Withdrawal of Consent	12 (4.63%)
Lost to Follow‐up	23 (8.88%)
Other	1 (0.39%)
Price	0
Lack of Efficacy	0
Have Reached Target Weight	0
Personal Reason	1 (0.39%)
Treatment Discontinuation: Yes	162 (62.55%)
Intolerant Adverse Event	8 (3.09%)
Other	154 (59.46%)
Price	78 (30.12%)
Lack of Efficacy	32 (12.36%)
Have Reached Target Weight	7 (2.70%)
Personal Reason	39 (15.06%)

**TABLE A2:** Changes in body weight in adults treated with liraglutide by sex.

		Baseline		Week 13 ± 5		Week 26 ± 5	
		Female (*N* = 112)	Male (*N* = 73)	Female (*N* = 97)	Male (*N* = 64)	Female (*N* = 33)	Male (*N* = 27)
Body weight (kg)	Mean ± SD	83.0 ± 12.1	102.6 ± 16.3	78.2 ± 11.4	96.7 ± 16.4	76.6 ± 10.7	94.7 ± 18.1
	Median (min, max)	80.5 (64.9, 125.0)	101.2 (75.9, 162.1)	76.2 (57.5, 118.7)	94.1 (74.5, 155.5)	76.7 (56.9, 104.6)	90.1 (72.5, 149.3)
Body weight loss (%)	Mean ± SD			−6.4 ± 4.0	−6.1 ± 3.4	−10.4 ± 5.7	−7.6 ± 5.6
	Median (min, max)			−6.2 (−21.4, 1.0)	−5.8 (−17.8, 3.8)	−9.9 (−24.5, −1.8)	−6.1 (−22.0, 1.9)
	*p*‐value^a^			*p* = 0.6671		*p* = 0.0576	

Abbreviations: max, maximum; min, minimum; SD, standard deviation.

^a^ *p*‐values were calculated by two‐sample *t*‐test.

**TABLE A3:** Changes in body weight in adults treated with liraglutide by age.

		Body weight (kg)		Body weight loss (%)		
		Mean ± SD	Median (min, max)	Mean ± SD	Median (min, max)	*p*‐value^a^
Baseline	20‐29 years (*N* = 21)	96.2 ± 19.9	91.6 (69.8, 137.0)			
	30–39 years (*N* = 51)	91.3 ± 14.4	88.8 (68.0, 131.5)			
	40–49 years (*N* = 53)	93.7 ± 16.6	88.0 (68.1, 145.7)			
	50–59 years (*N* = 45)	86.0 ± 18.7	80.0 (64.9, 162.1)			
	≥ 60 years (*N* = 15)	84.6 ± 11.3	84.0 (68.8, 102.5)			
Week 13 ± 5	20–29 years (*N* = 19)	89.1 ± 18.3	84.0 (59.6, 130.8)	−6.3 ± 5.5	−5.6 (−21.4, 1.0)	*p* = 0.9064
	30–39 years (*N* = 44)	85.4 ± 13.7	82.8 (63.3, 125.0)	−6.8 ± 3.3	−6.1 (−15.4, −2.3)	
	40–49 years (*N* = 43)	88.7 ± 16.3	84.6 (62.7, 144.7)	−6.1 ± 3.2	−6.6 (−16.0, 0)	
	50–59 years (*N* = 40)	82.7 ± 19.2	77.3 (57.5, 155.5)	−6.1 ± 4.2	−4.9 (−19.6, 3.8)	
	≥ 60 years (*N* = 15)	79.5 ± 10.9	78.5 (63.6, 97.5)	−6.0 ± 2.2	−6.2 (−9.9, −1.3)	
Week 26 ± 5	20–29 years (*N* = 7)	98.9 ± 19.1	96.9 (71.9, 134.0)	−8.4 ± 6.9	−5.9 (−22.0, −2.2)	*p* = 0.5603
	30–39 years (*N* = 17)	86.8 ± 14.9	82.9 (67.0, 121.0)	−7.4 ± 4.8	−6.9 (−18.7, −0.5)	
	40–49 years (*N* = 15)	80.2 ± 9.2	81.4 (59.2, 95.1)	−9.6 ± 6.5	−8.2 (−21.4, 1.9)	
	50–59 years(*N* = 14)	82.6 ± 23.4	79.2 (56.9, 149.3)	−10.8 ± 6.6	−8.5 (−24.5, −3.0)	
	≥ 60 years (*N* = 7)	79.4 ± 12.7	75.2 (60.9, 95.0)	−9.8 ± 3.7	−10.5 (−15.0, −4.0)	

Abbreviations: max, maximum; min, minimum; SD, standard deviation.

^a^ *p*‐values were calculated by ANOVA.
